# Mitochondrial DNA in forensic use

**DOI:** 10.1042/ETLS20210204

**Published:** 2021-08-10

**Authors:** Denise Syndercombe Court

**Affiliations:** King's College London, London 164651, U.K.

**Keywords:** ancient DNA, forensic, mitochondria

## Abstract

Genetic analysis of mitochondrial DNA (mtDNA) has always been a useful tool for forensic geneticists, mainly because of its ubiquitous presence in biological material, even in the absence of nuclear DNA. Sequencing, however, is not a skill that is part of the routine forensic analysis because of the relative rarity of requests, and the need for retention of necessary skill sets and associated accreditation issues. While standard Sanger sequencing may be relatively simple, many requests are made in the face of compromised biological samples. Newer technologies, provided through massively parallel sequencing (MPS), will increase the opportunity for scientists to include this tool in their routine, particularly for missing person investigations. MPS has also enabled a different approach to sequencing that can increase sensitivity in a more targeted approach. In these circumstances it is likely that only a laboratory that specialises in undertaking forensic mtDNA analysis will be able to take these difficult cases forward, more so because reviews of the literature have revealed significantly high levels of typing errors in publications reporting mtDNA sequences. The forensic community has set out important guidelines, not only in the practical aspects of analysis, but also in the interpretation of that sequence to ensure that accurate comparisons can be made. Analysis of low-level, compromised and ancient DNA is not easy, however, as contamination is extremely difficult to eliminate and circumstances leading to sequencing errors are all too easily introduced. These problems, and solutions, are discussed in the article in relation to several historic cases.

## Introduction

Mitochondrial DNA is found within membrane-bound organelles (mitochondria) in the cytoplasm of human nucleated cells where they generate most of the cell's supply of adenosine triphosphate (ATP). Mitochondria share many characteristics with bacteria and it is thought they were originally prokaryotes, becoming endosymbionts with eukaryotic cells [1]. The number of mitochondria within a single cell varies, depending on cell function, but range from none in a mature red cell through to many hundreds of thousands in an oocyte, while a human liver cell carries around 2000 [2], making mitochondria one of the main targets in forensic investigations when nuclear DNA is absent, limited or degraded.

**Figure 1.**
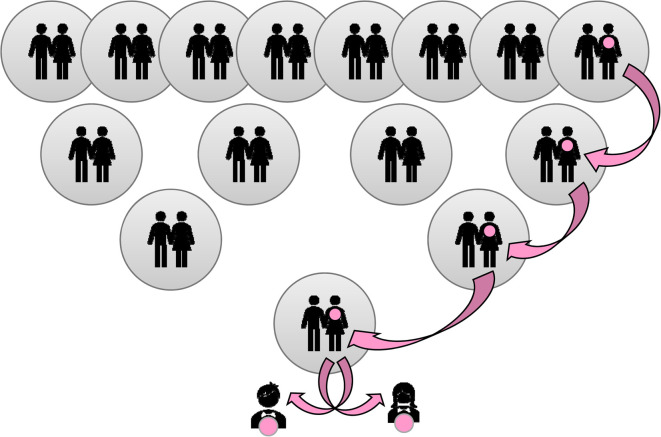
Illustration of MtDNA inheritance over four generations indicates ancestral females that share the same MtDNA haplotype as the children at the base.

Human identification for forensic purposes is defined by a set of autosomal genetic markers, in the form of short tandem repeats (STRs) that provide a virtually unique combination. These are small blocks of mainly four nucleotides repeated in sequence. The number of the repeats defines the allelic type. The mtDNA sequence, in contrast, refers not to an individual, but a group of individuals in the same maternal lineage, passed down from mother to child, as illustrated in Figure 1.

## Mitochondrial genome

Mitochondria have their own genome, not dissimilar to the genome of bacteria, and arranged in a histone-free, double-stranded, circular construction, with each mitochondrion having ∼2–5 whole-genome copies.

The mitochondrial genome was first fully sequenced in 1981 [3]; the Cambridge Reference Sequence, often referred to as the Anderson sequence, was later reanalysed to confirm and correct the sequence [4] with some conventions being maintained in order to maintain historic numbering. For convenience sequences are described as differences from the revised Cambridge Reference Sequence (rCRS) as published in GenBank NC 012920 [5].

MtDNA consists of both heavy (H) and light (L) strands, the density depending on the relative nucleotide distribution in each area. These regions represent most of the mtDNA molecule and consist of 37 genes coding for rRNA and tRNA, and thirteen polypeptides with most of the products supporting oxidative phosphorylation. Intron-less genes, seen in the mtDNA genome, is a characteristic feature of prokaryotes supporting the theory of bacterial origin; if removed from the cell, human nuclear DNA cannot regenerate the mitochondria.

Completing the rest of the molecule is a region which is a non-coding stretch of 1121 base pairs comprising the D-loop and a transcription promoter region — the ‘control region’. The D-loop consists of three strands of DNA, one complementary to one of the strands, holding it apart forming the displacement (D) loop [6].

**Figure 2.**
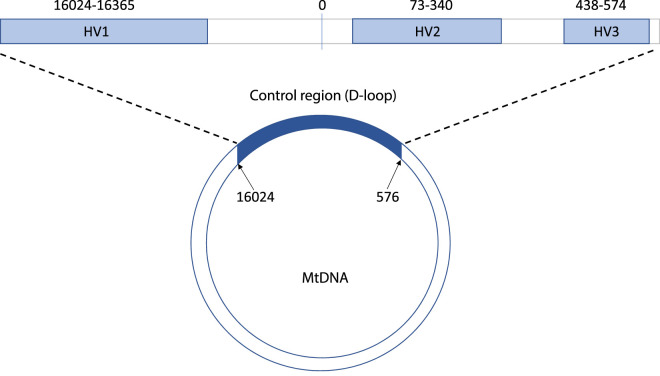
Diagram of the basic structure of the mitochondrial genome to highlight the hypervariable regions (HV1, HV2 and HV3) that lie within the ‘control’ or D-loop area of this genome that are of particular interest to forensic scientists.

According to the rCRS sequence in which each nucleotide is given a number, the complete control region lies between base pair positions 16 024 and 576. The nucleotide numbering extends from an MboI digestion point within the control region through into the L region [4,7], continuing round the molecule for a less variable 16 569 base pairs. Most sequence variability in the mtDNA genome is found in three areas within the control region, termed the hypervariable (HV) regions. Extending from 16 024 to 16 365 lies the HV1 region, from 73 to 340 lies HV2 and positions 438 to 574 define the HV3 region with more variability found in HV1 and HV2 than HV3 [8], explaining why the former two areas are the most commonly sequenced regions in forensic analysis (Figure 2).

## Mitochondrial sequencing

### Sanger sequencing

‘Sanger’ sequencing was developed from a primer-extension strategy to produce a rapid sequencing method involving chain-termination inhibitors that is still widely used today because of its ease and reliability [7]. Methodological improvements over time have led to this technology being available to forensic laboratories for many years, enabling sequencing of between 300 and 1000 nucleotides in one reaction.

The method requires a single-stranded DNA template, a DNA primer, DNA polymerase deoxynucleotidetriphosphates (dNTPs) and dideoxynucleotidetriphosphates (ddNTPs) that are labelled with different fluorescent dyes. The ddNTPs lack a 3′-OH group which prevents the formation of a phosphodiesterase bond between two nucleotides, terminating the extension when bound and causing the emission of light of a specific sequence dependent wavelength; electrophoretic separation differentiates and determines the sequence.

### Massively parallel sequencing

Massively parallel sequencing (MPS) (or next-generation sequencing (NGS)) has been commercially available since around 2005 and has superseded Sanger sequencing enabling sequencing of millions to billions of short reads in a single run, although the run length (up to 400 nucleotides) is shorter than Sanger sequencing. Many different MPS platforms have been developed to address sequencing in the medical diagnostics and pharmaceutical industries. This technology has not been employed extensively in forensic typing to date, although two commercial providers have produced MPS instruments dedicated to forensic requirements.

**Figure 3.**
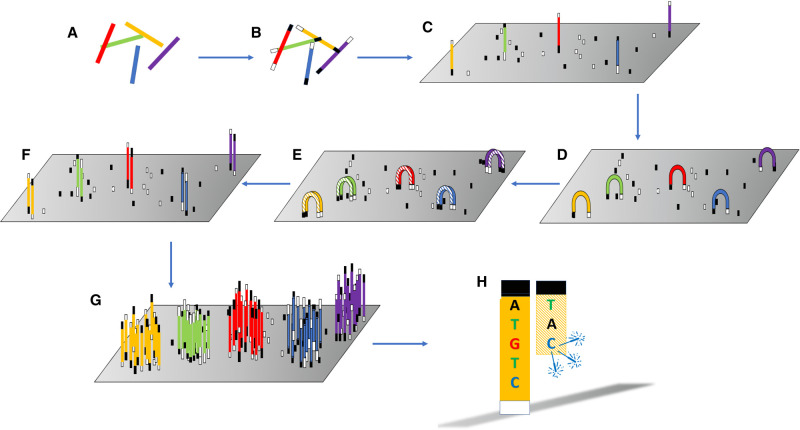
Principles of Illumina sequencing. (**A**) fragmentation; (**B**) Adapters annealed; (**C**) Fragments bind to glass surface loaded with complementary primers; (**D**) Bridge formation; (**E**) Bridge amplification; (**F**) Dissociation; (**G**) Cluster formed from repeated bridge formation and amplification; (**H**) Sequencing by synthesis and fluorescence detection.

**Figure 4.**
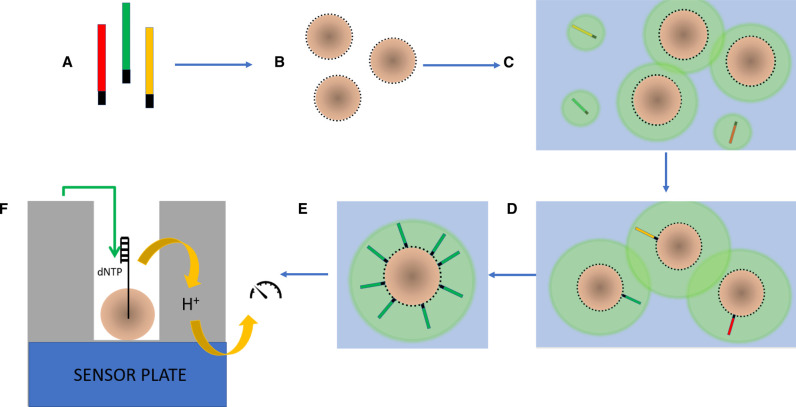
Principles of ThermoFisher Ion Torrent sequencing. (**A**) Adaptors annealed to fragmented library; (**B**) Beads prepared with primers attached to surface; (**C**) Beads and library mixed in an aqueous/oil mixture; (**D**) Dilutions prepared so that only a single fragment anneals to a single bead; (**E**) Standard PCR cycling on the emulsion enables clones of amplified bead-bound oligonucleotides to be developed with up to 100 000 copies on each bead; (**F**) Detector chips with millions of wells, each holding a single bead, enables massively parallel sequencing of the bound oligonucleotides within the well. Different dNTPs are introduced to the chip in sequence and when one binds to a complementary nucleotide a hydrogen ion is released, resulting in a change of pH.

MPS sequencing differs from the chain-termination method used by Sanger. The process is a miniaturised one that produces a ‘library’ of clonally amplified, bound, spatially separated, single-stranded fragments of the target molecule that are then sequenced by the addition of complementary nucleotides, simultaneously. Two bench top sequencers are common in forensic genetics: the Illumina MiSeq FGx, and the ThermoFisher Ion Torrent PGM and Ion S5 instruments. Both instrument types use a sequencing-by-synthesis approach in which a polymerase is used along with a signal, such as a fluorophore (used by the MiSeq instrument) (Figure 3) or change in pH (used in the Ion Torrent instrument) (Figure 4) to identify the incorporation of a nucleotide.

MPS technology offers cost-effective sequencing of the whole mitochondrial genome (mitogenome) with the provision of increased discrimination and phylogenetic resolution along with an assessment of data quality. Several groups have validated the different methodologies [9–11]. Missing person identification frequently makes use of mtDNA analysis, often because of the degraded nature of the remains, with significantly increased success rates when using MPS compared with Sanger sequencing [12].

In addition to improving discrimination power, the increased sensitivity of MPS can also reveal low-level variants not seen using Sanger sequencing but care must be taken to distinguish these from background noise and low-level contamination from intrinsic and extrinsic sources. The extent of the data produced with MPS means that robust bioinformatic approaches are required when mixtures are observed to validate observations of heteroplasmy [13].

## Mitochondrial mutation

The mitochondrial genome has a higher mutation rate in comparison with the nuclear genome (∼100 to 1000 times higher). Published mitochondrial DNA mutation rates are very variable, possibly reflecting the size of the individual studies, but also relate to how the rate is measured, through phylogenetic inference or through direct counting empirical experiments which have been made more possible in the advent of high throughput sequencing. In an Icelandic study [14] a rate of 0.0043 per generation compares with an estimate of 0.030 in another publication [15], although the latter includes mutations observed in poly C-tract. A meta-analysis combining eleven pedigree-related studies estimated the rate per generation at 0.0106 [16]. While phylogenetic-based mutation rates quoted are often much lower, using ‘relatively young’ ancient DNA studies of humans provides estimates only about two-fold lower than those calculated from pedigree observations. The contrasting published phylogenetic rates, sometimes ∼10 times lower, are thought to be related to different estimates that consider more recent human demographic histories of serial bottlenecks and expansions [17].

Although the lack of mtDNA repair mechanisms has often been cited as the reason for the relatively high mutation rate in comparison with nuclear DNA, the answer is much more complex and unclear. Research has revealed multiple repair pathways such as single strand break repair and mismatch repair, all apparently encoded by nuclear genes [18] and an interesting study revealed that the abundant mitochondrial dGTP in most tissues potentially leads to a loss of proof-reading activity by the exonuclease domain of the POLG (DNA polymerase subunit gamma) protein that removes DNA base-pair mismatches in replication [19].

### Heteroplasmy

**Figure 5.**
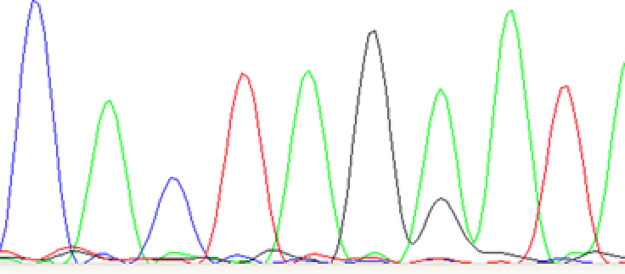
Sanger sequencing electropherogram of a sample showing point heteroplasmy in the 7th base from the left which shows two peaks coinciding representing bases A (in green) and G (in black). C and T bases are represented in blue and red, respectively.

Normally all the mtDNA in a cell has the same sequence (homoplasmy). Mitochondrial dysfunction due to mutations can impact on bioenergetic genes; those that are passed to the next generation can lead to severe inherited disease. Maintenance of the homoplasmic state is therefore very important but the mechanism poorly understood. Evidence points to some form of genetic bottleneck so that the mature oocyte represents a single molecule, which is generally free of mutations [20]. However, because mitochondria contain many mtDNA molecules, and each mitochondrion can have several genomes, it is not uncommon for mutations to be present affecting just a proportion of the molecules in the cell. Heteroplasmy is an example of a point mutation where a single base is substituted in portion of the genomes, demonstrating two different bases in the same position in the sequence (Figure 5). Clinically, there appears to be a threshold effect of the proportional change from wild to mutant type in a particular tissue when these mutations impact on the health of the individual.

Somatically, the level of heteroplasmy can be different in the different cells from the same tissue or organ, or between organs from the same person, or between individuals of the same family because different tissues have different bioenergetic thresholds [21].

Of forensic importance, variability in heteroplasmy can be seen between hairs, even from the same individual, probably relating to the developmental origins of individual hair follicles where there is localised cell division, in comparison with the lack of variability in blood cells in which the measured heteroplasmy will reflect the average seen across many stem cells. Rarely there may be a discordance between the haplotypes from a single hair root and from blood in the same individual with no demonstrable heteroplasmy; analysis of additional hairs should be undertaken in these very unusual circumstances [22].

## Mitochondrial inheritance

Mitochondrial DNA is considered to be inherited through the maternal line such that an individual's mtDNA will be shared with that person's mother and all prior maternal ancestors. It was originally thought likely that this maternal inheritance arose from a dilution effect, with mitochondrial DNA from the ovum outnumbering sperm mitochondrial DNA ∼100-fold, but research in algae [23] and fish [24] suggests that there may be an active process eliminating paternal mitochondrial DNA in order to preserve the health of this genome, as alluded to above.

Luo in 2018 raised the possibility of biparental mtDNA inheritance [25] when they reported three families with unusually high heteroplasmy, stating that the patterns could be explained by a combination of mtDNA from both parents. This hypothesis significantly challenged a long-held biological tenet and has led to both supporting [26] and critical responses [27,28], the latter making relevant comments about the methodology and the importance of confirmation in experienced and fully independent laboratories. In a recent study involving 11 035 mother-father-child trios [29], seven showed the same characteristics reported by Luo that, on face value, supported the hypothesis of paternal mtDNA inheritance. An alternative hypothesis is that it is due to the presence of nuclear-mitochondrial DNA segments (NUMTs) that mimic heteroplasmy. In each of the families at least one novel apparent NUMT was seen in the fathers, absent from the mothers. The observation of two full mitogenomes in an individual investigated for a possible contamination event revealed a megaNUMT in eight maternally related members of the family, in combination with the expected mtDNA haplogroup throughout the pedigree. The megaNUMT was absent from hair shaft, where nuclear DNA will be missing, offering a mechanism to investigate future claims of paternal mitochondrial inheritance [30].

## Mitochondrial variation in human populations

Mutations in mitochondrial sequences occur continuously and will affect the germline on occasions resulting in a heteroplasmy that is transmitted through the proposed genetic bottleneck. Over time, through genetic drift, the wild:variant balance can alter and when the sequence change is agnostic, or beneficial to the individual, can become fixed (homoplasmic) in the population.

### Mitochondrial haplogroups

The uniparental inheritance and lack of recombination has led to mitochondrial variants being restricted to particular populations in different parts of the world. There are varying definitions of the terms ‘mutation’, ‘polymorphism’, ‘single nucleotide polymorphism (snp)’, ‘point-mutation’ and the contexts in which they are used. Here we define snps as variants in a population with a frequency of 1% or higher [31], inferring that it is a polymorphism that is naturally occurring with a neutral or beneficial effect.

Different mitochondrial types, or haplogroups, are described according to a theoretical phylogenetic tree that offers evidence as to how these have evolved from a series of common ancestors. Understanding the nature of these variants has enabled population geneticists to track the matrilineal inheritance of humans back to an origin in Africa and has helped describe the spread of humans across the world.

**Figure 6.**
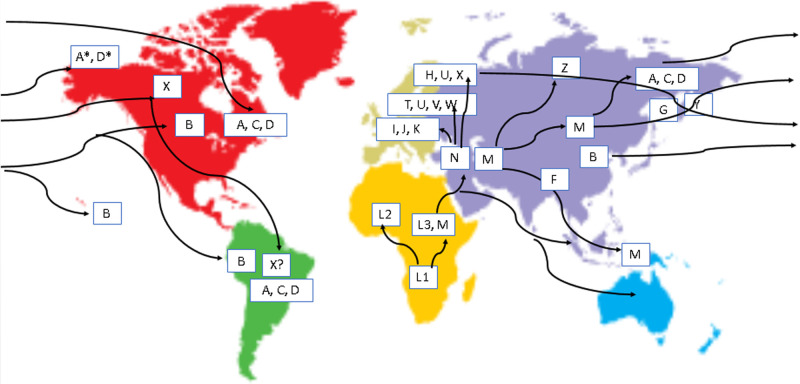
Major mtDNA haplogroups across the world and probable migration routes.

The major haplogroups have been named in letter order, A to Z, according to their discovery, with the hypothetical root (most recent common ancestor) that can be determined being referred to as ‘Mitochondrial Eve’, emerging ∼120 000 to 156 000 years ago and representing an individual from whom all living today have evolved, while not being the ‘first’ or ‘only’ woman of the species. Mitochondrial Eve belonged to haplogroup L. The main haplogroups and likely migration routes over time are illustrated in Figure 6. The phylogenetic tree is continuously updated [32] to reflect new information at www.Phylotree.org and uses the maximum parsimony approach [33] to define the optimal tree from the data.

## Forensic uses of mitochondrial DNA

MtDNA sequencing provides an additional useful tool to characterise biological evidence. While its ability to identify an individual is limited because of the lack of recombination, it offers significant advantages when confirmation of maternal lineage is sought or where nuclear DNA is limited such as in the analysis of bones, teeth and hair, because of its high copy number. Understandably it is often used in the analysis of ancient DNA and in the triage of disaster victim identification (DVI).

### Mitochondrial haplogroup frequencies

The rarity of a haplotype can be determined by simple counting of how many times the sequence is observed amongst samples of a database. As there may be various databases, both their representativeness and the quality of the sequences then becomes very important.

#### EDNAP mitochondrial DNA database (EMPOP)

EMPOP is probably the most comprehensive mtDNA database worldwide and forensically the most useful, not only because of the large number of world populations that are represented, but also because of its emphasis on high-quality data. The project was initiated through the European DNA Profiling Group (EDNAP) [34] and provides a freely accessible website (www.empop.online) which not only serves as a reference population database but all contributions have to go through quality assessment filters (EMPcheck and Quasi-Median Network tools) to detect sequence plausibility and transcription errors before the data is incorporated. EMPOP uses a string-based search algorithm (SAM2) [35] that converts sequences into alignment-free strings to ensure that haplotypes are properly defined regardless of alignment differences. In addition to providing the haplogroup, the tool clarifies nomenclature in the presence of phylogenetically unstable positions and takes block indels into account. The importance of quality assessment of mtDNA databases has been highlighted by several [36–38], revealing errors ranging from ∼10% to over 50%, many in medically focussed publications, and EMPOP aims to help address these problems.

### Forensic mitochondrial DNA typing

Superficially straightforward apparent differences in nomenclature may lead to false exclusions and a defined reporting process is vital. While both point heteroplasmy (discussed above) and length heteroplasmy are associated with mtDNA typing, reporting the latter is not required in forensic typing and it has no impact on the haplogroup. Samples with point heteroplasmy differences do not necessarily provide exclusionary evidence and sequences with a one-base difference should also be evaluated further with regard to their rate of mutation. Guidelines for forensic mtDNA typing have been produced by the International Society for Forensic Genetics (ISFG) and the Scientific Working Group on DNA Analysis Methods (SWGDAM).

#### SWGDAM guidance

The guidance highlights the importance of controls and avoidance of contamination, although they recognise that in mitochondrial analysis low-level contamination is difficult to avoid and suggest validated thresholds. Considering nomenclature, they recommend a blended application of rule-based and phylogenetic approaches and the use of the EMPOP database, although they advocate caution when considering historic data which might have been interpreted differently. Rules are provided to advise about homopolymeric C-stretches, particularly important in MPS workflows, and they provide nomenclature recommendations on substitutions and indels, transitions and transversions [39].

Reporting guidance suggests the use of three interpretive categories, with two or more nucleotide differences (excluding length heteroplasmy) providing evidence of an ‘exclusion’; ‘inconclusive’ if there is one nucleotide difference; ‘cannot exclude’ being the third category. Frequency reporting is done with simple counting in an appropriate recognised population database. SWGDAM plan to review these guidelines to address whole mitogenome reporting.

#### ISFG guidance

An updated series of thirteen recommendations is provided by the ISFG to supplement that provided in 2000. Like SWGDAM they highlight methods to avoid errors and deal with potential low-level contamination, with recommendations for independent confirmation and participation in appropriate proficiency tests. They continue to recommend reporting with reference to the rCRS reference sequence but with the addition of information on the sequencing range used, while recommending that the complete mtDNA control region should be typed. Guidance and tools for nomenclature decisions are also provided [40,41].

Forensic laboratories are advised to establish their own interpretation and reporting guidelines for both length and point heteroplasmy because of the differences in technologies now being used with a choice as to whether length heteroplasmy is reported. Because of the high incidence of mtDNA sequence interpretation errors [38] they recommend the use of software quality tools to check that the phylogeny is as expected. Homopolymeric C-tracts are avoided by default in EMPOP and should also be ignored if other databases are searched.

Use of alignment-based database searches, facilitated through EMPOP, of the whole database is recommended to avoid alignment differences and reporting bias. The database used to assess the significance of a match should be considered, relevant to the case circumstances. Different frequency estimates can be considered, such as using the conservative upper 95% confidence interval. Whatever is chosen should be able to be justified considering any uncertainties, taking into account the fact that mtDNA frequencies may vary significantly at a local population level [42].

## Ancient and degraded DNA

Techniques developed to analyse ancient DNA can provide useful approaches to sequence significantly degraded material associated with a crime scene. Bones must be carefully and thoroughly cleaned to remove external contaminants and the analysis undertaken in laboratories dedicated to the analysis of extremely low-level DNA.

Over time DNA degrades and can produce false sequences. Hydrolytic damage can lead to sequence miscoding through deamination. Deamination of cytosine produces uracil, which pairs with adenine producing thymine through PCR; miscoding produces the majority of sequencing errors. Treatment with N-glycosylase to remove uracils can assist here [43]. Another problem can be unexpectedly long chimeric sequences introduced through PCR of degraded DNA fragments due to ‘PCR jumping’ in which an additional adenosine molecule may be added to the end of a DNA template and this then ‘jumps’ to another template — polymerisation continues, creating an *in vitro* recombination product. This is particularly problematic if the amplification is initiated only from a few copies and suspicion should be raised if there are unexpectedly successful products. Contamination with modern DNA and bacterial DNA adds further to the problems in analysis of degraded DNA

When sample quality is an issue a capture-hybridisation method [44] can be used to enrich the mtDNA. NUMTs, however, will also be enriched and the target sequence may be misinterpreted as a heteroplasmic sequence. Removal of known NUMTs and application of validated filters to deal with others will considerably decrease sequencing errors [45].

## Use of mitochondrial DNA in cases of historical interest

Analysis of mitochondrial DNA can provide useful additional information to support poor or negative autosomal information in criminal cases and can be particularly helpful in the analysis of skeletal material, hairs without roots, or severely burnt remains. Mitochondrial DNA can also assist when triaging remains in DVI situations. Mitochondrial DNA analysis has played a significant role in the understanding of population movements and domestication, and in the investigation of mass graves associated with past conflicts. Occasionally it can be used to answer questions of historic interest and some of these are described below; each highlights important issues arising from the identification.

### Louis XV11 (King of France 1793–1795)

Louis Charles was just eight when he inherited the throne of France but died shortly after. Following royal tradition, the boy's heart was placed, but not until 1975, in the Basilica in Paris where members of the French Royal Family are buried.

Many, however, believed that his body had been substituted with another. Many individuals claimed Louis XVII as their direct relative; prominent amongst them was a German clockmaker but in 1998 comparisons of MtDNA showed no match [46]. The question remained, however, as to whether this relic was from Louis XVII.

MtDNA analysis of HV1 and HVII provided a consensus sequence that matched with maternally related living Habsburg descendants [47], providing a D-loop sequence not seen in 1700 Europeans offering support to the identification.

This study illustrates the importance of adhering to strict guidelines in the analysis of ancient DNA. The authors had followed these, and the heart samples were analysed by two different institutions. Many of the fragments sequenced were short, as was expected, and all reproducible, whereas attempts to analyse longer sequences produced different results. Minor contamination associated with PCR artefacts such as PCR jumping could have led to preferential amplification of these erroneous longer sequences.

### Sinking of RMS titanic (15 April 1915)

Just over 30% of the passengers of the Titanic survived when it was sunk after hitting an iceberg. A young child (the ‘Unknown Child’) was long believed to be a Gösta Pålsson based on contemporaneous evidence.

A project in 2001, to confirm the identification using mtDNA analysis of HV1, could not confirm the match but did accord with the families of two other missing children — Eino Panula and Sidney Goodwin. Odontological age determination favoured the former when reported in 2004; doubts continued because of non-matching shoe sizes. Extended control region sequencing revealed two sequence differences from the Panula references, and the unknown child is now expected to be that of Sidney Goodwin [48].

The misidentifications highlight the difficulties associated with DVI in which material may be limited, degraded and potentially contaminated, and especially when living families have to balance their desire and expectation of a rapid resolution and the lengthy and complex scientific analysis that may be required for as secure as possible identification.

### Identifying the Romanov family

The Russian Imperial Royal family members (the Emperor, the Empress and their five children) were assassinated in July 1918 and buried in the Koptakyi forest until skeletal remains were found in 1979 and more later in 2007. DNA tests were commissioned to be conducted by Russian and UK geneticists [49]. Mitochondrial DNA (HVI and HVII) was used to link the putative remains of the Tsarina to a distant maternal living relative, Prince Philip, giving a complete match. Analysis of the femur of the Tsar, Nicholas II, however, revealed a C/T heteroplasmy at position 16 169 which was not seen in living relatives, who were homoplasmic T. The heteroplasmy was, however, verified when the body of the Tsar's brother, Georgii was exhumed. Further confirmation of the original identification was made from clothing worn by Nicholas II at the time of his death. Challenges were made to the identification with a scientist claiming non-matching results from a handkerchief stained with blood from Nicholas II (unpublished), and absent heteroplasmy in preserved hair from Georgii; publication of the sequence they obtained (as reported by Coble in his comment piece [50]) however, revealed that these samples were almost certainly contaminated. An additional criticism of the positive identification came from analysis of a finger bone purportedly from the Tsarina's sister, which the scientist claimed did not match with the haplotype of Prince Philip. The authors criticised the nested approach used by Gill claiming that this had led to an erroneous match, however their own analysis revealed a mixture and they had used a consensus sequence to report their non-match conclusion [51].

The various reports initiated much comment in the literature and they highlight the importance of independent analysis, replication of results, validation of methods, genetic consistency and verifiable reference material, all of which was absent from the work of the several critics.

## Summary

- Mitochondrial DNA analysis offers a significant advantage to the forensic geneticist in circumstances when it has not been possible to obtain a standard nuclear DNA profile, such as in severely degraded DNA, bones and hair shafts.
- Mitochondria are inherited through the maternal line and will be present in both male and female living children offering tools to assist in familial links over many generations, such as in missing person cases where there is not direct family reference.
- Sequencing of mitochondria requires a good knowledge of bioinformatic tools to ensure correct sequence and haplotype information.
- Ancient and degraded DNA provides many challenges in mitochondrial sequencing because of the competing presence of contaminants, amplification product verification challenges and interfering sequence copies from other parts of the genome, all requiring specialist provision and expertise.

## Open Access

Open access for this article was enabled by the participation of Kings College London in an all-inclusive *Read & Publish* pilot with Portland Press and the Biochemical Society under a transformative agreement with JISC.

## Abbreviations

ATP: adenosine triphosphate
ISFG: International Society for Forensic Genetics
MPS: massively parallel sequencing
NUMTs: nuclear-mitochondrial DNA segments
SWGDAM: Scientific Working Group on DNA Analysis Methods
